# Author response to “lack of benefit from low dose computed tomography in screening for lung cancer”

**DOI:** 10.1186/s12890-020-01247-y

**Published:** 2020-08-26

**Authors:** Kai-Lin Huang, Shih-Yuan Wang, Wan-Chen Lu, Ya-Hui Chang, Jian Su, Yen-Ta Lu

**Affiliations:** 1 Department of Pharmacy, MacKay Memorial Hospital, No. 92, Sec. 2, Zhongshan N. Rd, Taipei City, 10449 Taiwan; 2 Mackay Junior College of Medicine, Nursing, and Management, No.92, Shengjing Road, Beitou District, Taipei, 11272 Taiwan; 3 Department of Chest Medicine, MacKay Memorial Hospital, No. 92, Sec. 2, Zhongshan N. Rd, Taipei City, 10449 Taiwan; 4 Department of Medicine, Mackay Medical College, No.46, Sec. 3, Zhongzheng Rd., Sanzhi Dist, New Taipei City, 252 Taiwan

**Keywords:** Low-dose computed tomography, LDCT, Lung cancer screening, Mortality, Meta-analysis

## Abstract

We explain to Dr. Benjamin (corresponding author) about why low-dose computed tomography reduce lung cancer mortality without significantly reducing all-cause mortality. We also conduct an up-to-date meta-analysis to evaluate low-dose computed tomography clinical effectiveness compared with usual care of lung cancer screening.

## Author response

Dr. Benjamin points out that the inconsistencies between lung cancer mortality and all-cause mortality in our article [1] [low-dose computed tomography (LDCT) reduced lung cancer mortality (rate ratio (RR) 0.83, 95% confidence interval (CI) 0.76–0.90, I^2^ = 1%) but had no effect on all-cause mortality (RR 0.95, 95% CI 0.90–1.00)]. We agree and acknowledge in our abstracts. The data shows that all-cause mortality is moving in the same direction as lung cancer mortality (favor LDCT), even if statistical significance cannot be demonstrated. We also agree that mortality benefits of screening trial require cautious scrutiny. The accuracy of lung cancer mortality depends on identifying the cause of death accurately which may be subject to bias. Therefore, all-cause mortality should be reported and considered together with lung cancer mortality [2].

**Fig. 1:**
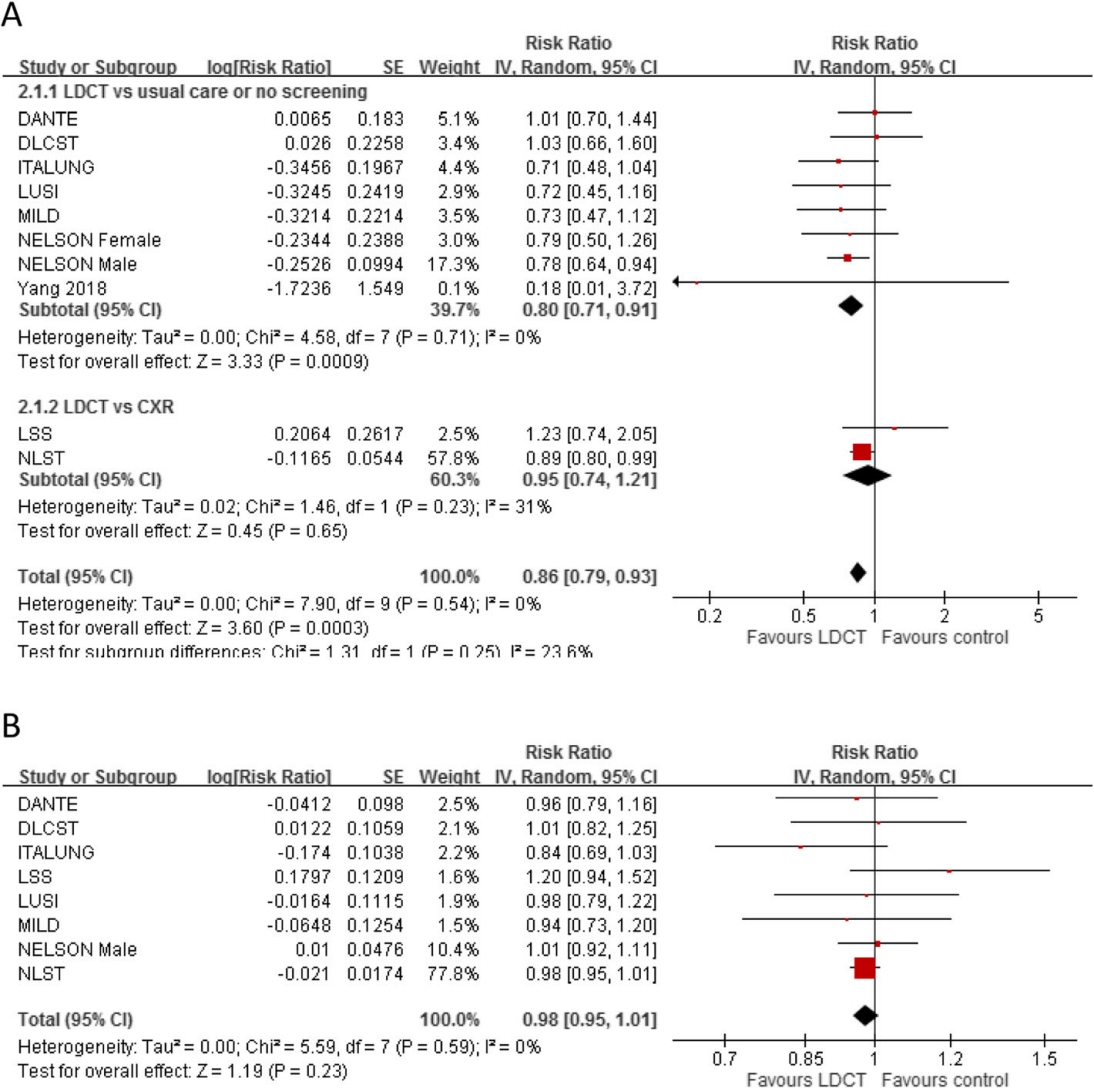
Forest plots of comparisons between low-dose computed tomography (LDCT) versus no screening or chest radiology (CXR) for (**a**) lung cancer mortality (**b**) all-cause mortality using update data

Three trials recently update their extended follow-up data [National Lung Screening Trial (NLST) [3], Nederlands–Leuvens Longkanker Screenings Onderzoek Study (NELSON) [4] and German Lung Cancer Screening Intervention Trial (LUSI) [5]]. We have re-analyzed the data by using the latest mortality rate (shown in Fig. 1). The lung cancer mortality rate ratio is 0.86 (95% CI 0.79–0.93, I^2^ = 0%). For all-cause mortality, rate ratio is 0.98 (95%CI 0.95–1.01). We also conduct sensitivity analyses to test the robustness of the results. The leave-one-out analysis is performed by omitting one study in turn. Even when we exclude NLST trial (which Dr. Benjamin think it might have potential methodological flaws), results are still robust. The outcome is consistent with our previous analysis. As suggested by Prasad et al. [6] and Dr. Benjamin, limited evidence shows that chest radiography (CXR) may increase death from lung cancer, in which some deaths are attributed to detected cancer incorrectly. Thus, no screening (usual care) would be a more appropriate comparator. In the subgroup from our analyses indicate that, compare with no screening (usual care), LDCT screening still has effect in reduction lung cancer mortality (RR 0.83, 95% CI 0.71–0.91).

There are several possible explanations why LDCT reduce lung cancer mortality without significantly reducing all-cause mortality. To begin with, some of the inconsistencies observed may be due to chance, in particular when lung cancer mortality is proportionally low. Pooling of the available data in a meta-analysis may solve the problem. Then, if the percentage of cancer deaths among all deaths is low, there isn’t much risk to reduce. Even for a useful cancer screening procedures, the effect would be small in absolute terms (in the case of NELSON, a 24% decrease in the relative risk of dying of lung cancer in men translated to an approximately 0.75% reduction in lung cancer mortality (from 3.18% in the control group to 2.43% in the LDCT group) after 10 years of follow-up) [4]. Thus, reduction in lung cancer mortality with LDCT screening is too low to considerably affect all-cause mortality. Furthermore, studies may not be sufficiently powered to detect a possible favorable difference in all-cause mortality. According to Heijnsdijk et al. [7], a significant reduction in all-cause mortality could be expected between 11 and 13 years of follow-up for a sample size of minimal 40,000 high risk participants in each arm. Studies included in our meta-analysis have sample sizes far below 80,000 and some of trials less than 11 years of follow up. One cannot expect statistically significant declines in all-cause mortality. Finally, subsequent deaths from cancers develop after the screening window and other causes of death appear [3]. It will lead to a trend towards a reduced difference between two groups and diminishing levels of significance. It would be unrealistic to expect a single intervention designed to contribute to reduced mortality from one cause to reduce mortality from all causes. After all, the primary aim of cancer screening is to prevent premature death from one or several related causes.

However, harms of future invasive procedures for diagnosis purpose, overdiagnosis and overtreatment cannot be fully accounted by using lung cancer mortality as outcome. All-cause mortality reductions may be offset by harms due to the LDCT screening. Although LDCT shows an increase in detection of early stage cancers, overdiagnosis and false positive test results may increase as well. These results in unnecessary diagnostic procedures and lead to unnecessary treatment. When the possible benefits and harms of each option affect patients differently, shared decision making is recommended. Besides, targeting high risk population and image analysis methods refinement may further improve the efficiency of LDCT screening.

Since lung cancer is the leading cause of cancer-related death among men and women, if it is found at an earlier stage, it is more likely to be successfully treated. Prevention (e.g. smoking cessation) is likely to have far greater impact on lung cancer mortality than is screening. Nonetheless, LDCT screening has the potential to significantly reduce the burden of lung cancer. From a public health perspective, 14% reduction in lung cancer mortality and 2% reduction in all-cause mortality (even though the effect is not statistically significant) may be relevant. More studies are warranted to optimize risk-stratified recruitment strategies and radiologic criteria.

## Data Availability

All data generated or analyzed during this study are included in this published article.

## Abbreviations

LDCT: Low-dose computed tomography
RR: Rate ratio
CI: Confidence interval
NLST: National Lung Screening Trial
NELSON: Nederlands–Leuvens Longkanker Screenings Onderzoek Study
LUSI: German Lung Cancer Screening Intervention Trial
CXR: Chest radiography
